# Confirming the factor structure of a generic quality of life instrument among pre-treatment substance use disorder patients

**DOI:** 10.1186/s12955-019-1152-7

**Published:** 2019-05-17

**Authors:** Ashley Elizabeth Muller, Svetlana Skurtveit, Thomas Clausen

**Affiliations:** 1 Norwegian Centre for Addiction Research, Institute of Clinical Medicine, University of Oslo, Pb 1039 Blinder, 0136 Oslo, Norway; 2 Department of Mental Disorders, Norwegian Institute of Public Health, Oslo, Norway

**Keywords:** Substance use disorder, Social quality of life, QOL10, WHOQOL-BREF, Structural equation modelling, Isolation

## Abstract

**Background:**

Quality of life (QoL) is a patient-reported outcome of increasing importance in the substance use disorder (SUD) treatment field, and impaired QoL may be an important impetus for treatment uptake. Instruments and methodologies abound, precluding comparison, as does a dearth of population norms. The QOL10 is a generic, overall QoL tool containing ten items and with simple scoring procedures. It is therefore a potential alternative to the gold standard WHOQOL-BREF. This study aimed to assess the two-factor structure of the QOL10 that has been suggested by a previous exploratory factor analysis.

**Methods:**

Adults entering 21 participating inpatient or outpatient SUD treatment were recruited to join a national longitudinal cohort study. 531 completed the QOL10 at treatment entry and were included in the analysis. Structural equation modelling (SEM) was used to confirm the model fit of a two-factor structure, and the scaling qualities of the QOL10 were reported.

**Results:**

According to the SEM analysis, the QOL10 was comprised of one latent variable measuring social QoL, and one measuring global QoL, and all ten items were retained. Goodness of fit tests included: root mean square of approximation = 0.063, 90% CI 0.050–0.076; normed-fit index = 0.919; and comparative fit index = 0.943.

**Conclusions:**

The QOL10 should be considered when clinicians in the SUD treatment field need a short, valid instrument that measures both global QoL and social QoL, with minimum respondent and administrator burden. The social domain is of particular utility and may be used as a stand-alone instrument. Test-retest reliability should be established in future studies.

**Electronic supplementary material:**

The online version of this article (10.1186/s12955-019-1152-7) contains supplementary material, which is available to authorized users.

## Background

Quality of life (QoL) is an operationalization of the recovery model of substance use disorders (SUD) proposed by Substance Abuse and Mental Health Services Administration [1], and as such is an important treatment outcome measure [2]. As a necessarily patient-reported measure [3], QoL captures the lived impacts of SUD and treatment on a person’s life, using information to which a physician or treatment provider is not privy [4]. The desire to improve QoL may be a more important motivation for treatment uptake than the reduction of substance use per se [5]. Given the wide-ranging social, medical, and legal consequences of SUDs, it is unsurprising that SUD patients have lower QoL than other chronic disease groups [6–8].

The International Society for Quality of Life Research suggests minimum standards for QoL instruments in addition to validity and reliability, such as a low burden to both respondents and administrators, i.e. using a minimum amount of items and simple scoring procedures [9]. Yet recent reviews of QoL measures among opioid users have highlighted that even existing instruments are being scored and presented differently [8, 10]. The current gold standard among SUD patients is the World Health Organization’s WHOQOL-BREF, a 26-item measure with four domains of physical health, psychological health, social relationships, and environment QoL [11]. Scoring instructions are not simple, however, and include syntax for the use of statistical software. A validation study reported that some of the negatively worded items may not have been understandable by substance users [12]. Another commonly used measure, the Lancashire Quality of Life Profile (LQoLP) [13], is psychometrically strong but imposes even more burden, with 133 items administered in a structured interview.

The generic, multidimensional QOL10 [14] may be a less burdensome and more acceptable alternative for the SUD population. The QOL10 has fewer items (ten) than the WHOQOL-BREF, no negatively worded items (compared to three), and the scoring procedure is simple. A previous analysis on a small sample demonstrated convergent validity to the WHOQOL-BREF and suggested that a two-domain structure of “social QoL” and “global QoL” was a better fit than the QOL10’s original hypothesized three-domain structure [15]. This previous analysis also reported that the QOL10’s social QoL domain had higher internal reliability than the WHOQOL-BREF’s social relationships domain. Exploration of the QOL10 with a larger sample size as well as more sophisticated analytic methods are needed.

This analysis confirms a two-domain structure of the QOL10 instrument, and reports on item responses, mean values, and scaling qualities. Data are drawn from 531 patients entering heterogeneous SUD treatment programs across Norway.

## Methods

### Design and setting

Data from this analysis is drawn from the longitudinal Norwegian Cohort Study of Patients in Opioid Maintenance Treatment and Other Drug Treatment (NorComt) Study. Participants were recruited to NorComt upon entry to any of the 21 participating treatment facilities, with no exclusion criteria, between 2012 and 2015. After collecting written informed consent, treatment providers who had been trained by the research team administered a 100-item questionnaire to entering patients. The questionnaire included the QOL10, described below, and other validated measures such as the Europe-ASI [16] and Hopkins Symptoms Checklist 25 [17]. Sixty to ninety minutes on average were spent filling out the questionnaire.

### Participants

Participant characteristics of the entire NorComt study population at treatment and study entry have been reported on previously [18, 19]. Briefly, 531 of 548 provided valid QOL10 scores (described in “Quality of life instrument” below) and were included in this analysis. The sample was comprised of 28.2% women (*n* = 150), with an average age of 33.4 (*SD* 9.8). Half had a substance-using social network (51.4%, *n* = 272) while 16.9% had no network (*n* = 90), and 55.9% (*n* = 297) reported over the cut-off for clinically concerning mental distress on the Hopkins Symptoms Checklist-25. Forty-four percent reported eating most of their meals alone (*n* = 235), while 53.1% ate with friends, family, or others (*n* = 282). Half were entering into outpatient opioid maintenance treatment (50.1%, *n* = 266), and half into residential treatment for opioid or other substances (49.9%, *n* = 265). Nearly all were polysubstance users (91.5%, *n* = 485), with the most commonly used substance in the past six months for a plurality being heroin (23.7%, *n* = 126), followed by amphetamines (19.8%, *n* = 105), cannabis (16.9%, *n* = 90), and alcohol (7.9%, *n* = 42).

### Quality of life instrument

The QOL10 includes ten items asking for participants’ current evaluation of various aspects of their life, such as “how is your working ability at the moment?” and “how are your relationships with your friends at the moment?” All items are answered on a 1–5 Likert-type scale from “very poor” to “very good”, with a neutral option. In a previous study of the QOL10 using a subsample of the first NorComt participants followed up with after one year, during 2013–2015, an exploratory factor analysis suggested that a two-factor structure of a *social QoL domain* and a *global QoL domain* was a good fit [15]. The social QoL domain contained five items (item 4: friends, 5: partner, 6: ability to love, 7: sexual functioning, and 8: social functioning), while the global QoL domain contained the remaining five items (item 1: physical health, 2: mental health, 3: feel about yourself, 9: work, and 10: overall QoL). Both social and global QoL domain scores were converted to 0–100 scales to be comparable with the WHOQOL tools [11], and both domains had acceptable internal consistency (α = 0.814 for social QoL, and α = 0.771 for global QoL) The WHOQOL Group suggests an upper limit of 20% missing data for the calculation of domain scores, therefore all five items in the global QoL domain were required. In the social QoL domain, only the partner item was allowed missing, to avoid excluding single participants, and the social QoL domain calculated based on the remaining four items. Seventeen participants were deleted listwise due to lacking one or both domain scores, leaving 531 participants in the analysis.

### Analysis

Structural equation modelling (SEM) is a sophisticated multivariate procedure that tests a priori relationships between observed and latent variables in a structural theory [20]. Utilizing a confirmatory factor analysis approach and maximum likelihood estimation procedure, SEM was used to assess a two-factor structure suggested by the earlier exploratory factor analysis [15], with the friends, partner, ability to love, sexual functioning, and social functioning observed variables loaded onto the latent variable *social QoL*, and the physical health, mental health, feel about yourself, work, and overall QoL observed variables loaded onto the latent variable *global QoL*. Covariance paths were entered between the two latent variables. Model fit was assessed by four indices, as suggested by Kline [21]: the model chi-square, a traditional measure of overall fit that is sensitive to sample size and assumes multivariate normality (*p* > 0.05 represents a good fit); two alternative indices to the chi-square, the normed-fit index, indicating the improvement in fit of the model of interest relative to the null model, and comparative fit index, a revised form of the normed-fit index that is less sensitive to sample size (values ≥0.90 represent a good fit); and the root mean square error of approximation, a parsimony-adjusted index in which lower values are preferable (values ≤0.05 represent a good fit, and confidence limit upper values should be ≤0.08), Hoelter’s statistic assessed the adequacy of sample size (≥200) [22]. All statistics were performed on SPSS AMOS v25.

## Results

Table 1 displays the scaling qualities of the QOL10. On a scale of 0–100, the mean social QoL domain was 53.5 (*SD* 20.0), and the mean global QoL domain was 32.9 (*SD* 19.1). Both domains were non-normally distributed due to positive skews, but examination of the normal Q-Q plots determined non-normality to be modest. Additional file 1: Table S1 displays the response distribution to each of the ten items, and Additional file 2: Table S2 displays item-total correlations to the two domain scores.

**Table 1:** Scaling qualities of the QOL10 domains

	Social QoL	Global QoL
Mean (*SD*)	53.46 (*20.02*)	32.93 (*19.09*)
Median (IQR)	55.00 (28.89)	30.00 (25.00)
Range	0–100	0–90
CI	51.64–55.32	31.38–34.63)
Kurtosis (SE)	−.48 (.21)	−.45 (.21)
Skewness (SE)	.01 (.11)	.35 (.11)
Significant test of normality^a^	< 0.001	<.001
Cronbach’s alpha	.707	.770

^a^One-Sample Kolmogorov-Smirnov test

**Fig. 1:**
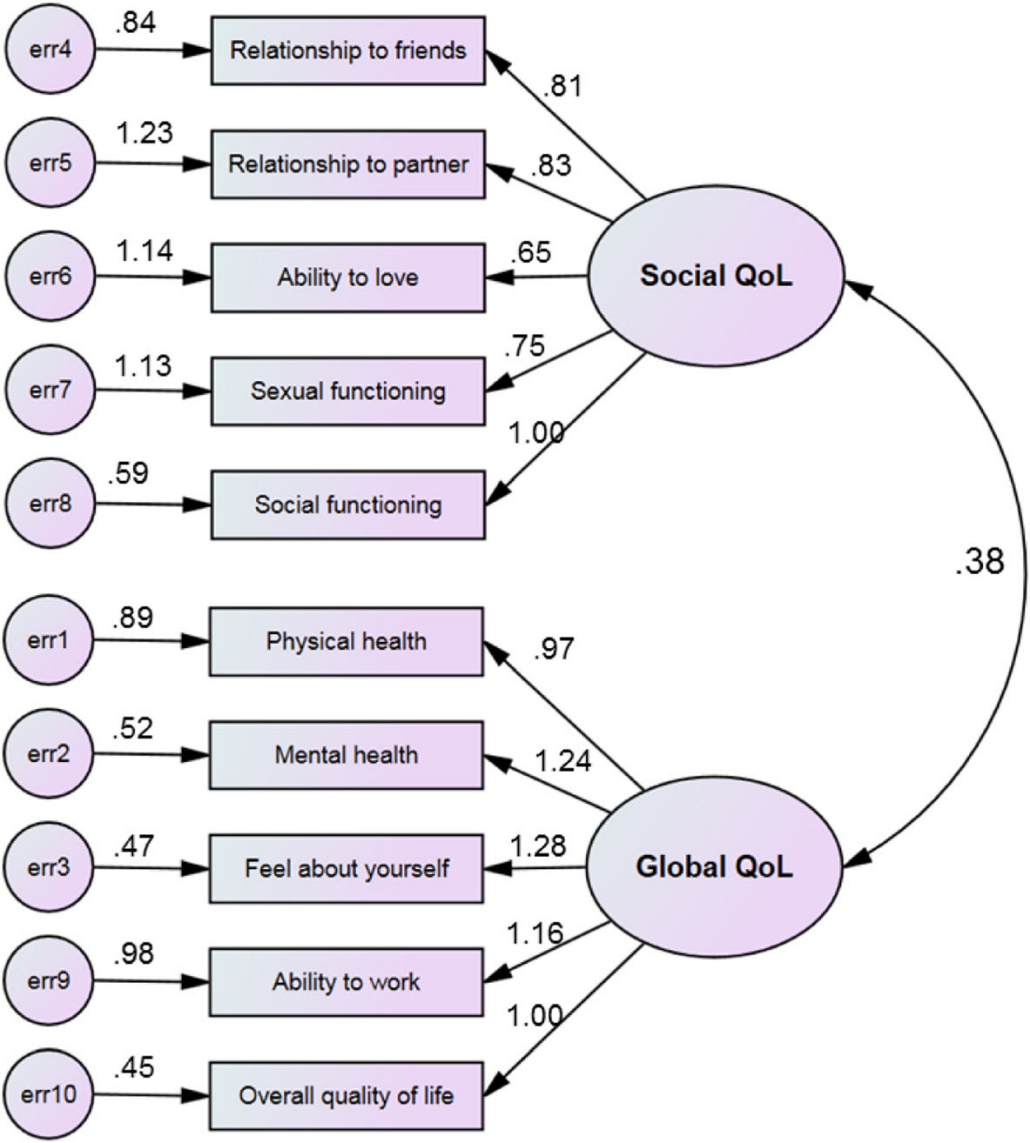
Structural model with parameter estimates of overall quality of life. According to the SEM analysis, the QOL10 was comprised of one latent variable measuring social QoL, and one measuring global QoL

The hypothesized relationships between the social domain and the global domain are contained in the structural equation model, displayed in Fig. 1. The two domains were positively related to one another, with a standardized regression coefficient of 0.38. The model chi-square = 107.2 with 34 degrees of freedom, *p* < 0.001; this poor fit was likely due to a large sample size and positively skewed domain scores. The remainder of the indices indicated that the model was a good fit: normed-fit index = 0.919; comparative fit index = 0.943; and root mean square error of approximation = 0.063, 90% CI 0.050–0.076. Hoelter’s test = 249, indicating an adequate sample size.

## Discussion

Using data from a large national treatment study of SUD in Norway, this analysis aimed to explore the factor structure of the generic QOL10 instrument administered to 531 patients entering a variety of substance use disorder treatments. The QOL10 was confirmed to measure social QoL and global QoL with five items in each domain and a stable factor structure [15]. The overall QoL of this sample in a previous study was found to be severely impaired [18], as has been extensively reported among this patient group [7, 8, 23]. Our sample’s social QoL (53) was slightly higher than their global QoL (33), a pattern also found in a large sample of opioid use disorder patients beginning treatment in Germany [24]. In the German study, a different instrument with the same scoring scale was used, and three social domain scores (psychosocial, partner, and family domains) ranged from 42 to 57, while the general QoL domain score was 38. These results should not be interpreted as this sample or others with substance use disorders having acceptable social QoL, rather, that their global QoL – accounting for health, working ability, and an overall QoL evaluation – is extremely impaired.

## Conclusions

The QOL10 is a valid instrument that could be considered an even shorter alternative to the gold standard WHOQOL-BREF, particularly when the social domain of QoL is of interest. The QOL10’s social domain had higher internal reliability than the WHOQOL-BREF’s corresponding domain [15], and by including two additional items, may collect more relevant information than the WHOQOL-BREF without significant extra burden. The social QoL domain of the QOL10 may also be extracted and used independently, a particularly helpful feature of this instrument given that the stigma associated with SUDs, including treatment-seeking, can reduce this domain [23, 25], while social support may protect it [26–28]. Attention to social QoL in a clinical setting could expose areas for intervention, such as social networks, a lack of abstinence-specific social support, or anti-stigma training among health care professionals. More research in general is needed on social QoL, and psychometrically strong instruments are the first step.

## Additional files

Additional file 1: **Table S1** QOL10 item responses among 531 patients entering substance use disorder treatment, N(%) (DOCX 14 kb)

Additional file 2: **Table S2** QOL10 item-total correlations (DOCX 14 kb)

## Abbreviations

NorComt: Norwegian Cohort Study of Patients in Opioid Maintenance Treatment and Other Drug Treatment Study
QoL: Quality of life
SEM: Structural equation modeling
SUD: Substance use disorder

## Authors’ information

AEM researches quality of life among substance-using and other marginalized populations. SS is a senior researcher in pharmacoepidemiology at the Norwegian Institute for Public Health and leads national and Nordic-wide registry-based projects. TC heads the Norwegian Centre for Addiction Research and leads the yearly evaluation of the national opioid maintenance treatment program.

## References

[1] Chiu MY, Ho WW, Lo WT, Yiu MG (2010). Operationalization of the SAMHSA model of recovery: a quality of life perspective. Qual Life Res.

[2] Worley J (2017). Recovery in substance use disorders: what to know to inform practice. Issues Ment Health Nurs.

[3] Rapkin BD, Schwartz CE (2004). Toward a theoretical model of quality-of-life appraisal: implications of findings from studies of response shift. Health Qual Life Outcomes.

[4] Laudet AB (2011). The case for considering quality of life in addiction research and clinical practice. Addict Sci Clin Pract.

[5] Laudet AB, Stanick V, Sands B (2009). What could the program have done differently? A qualitative examination of reasons for leaving outpatient treatment. J Subst Abus Treat.

[6] Vederhus J-K, Pripp AH, Clausen T (2016). Quality of life in patients with substance use disorders admitted to detoxification compared with those admitted to hospitals for medical disorders: follow-up results. Subst Abuse.

[7] Foster JH, Powell JE, Marshall EJ, Peters TJ (1999). Quality of life in alcohol-dependent subjects – a review. Qual Life Res.

[8] De Maeyer J, Vanderplasschen W, Broekaert E (2010). Quality of life among opiate-dependent individuals: a review of the literature. Int J Drug Policy.

[9] Reeve BB, Wyrwich KW, Wu AW, Velikova G, Terwee CB, Snyder CF, Schwartz C, Revicki DA, Moinpour CM, McLeod LD (2013). ISOQOL recommends minimum standards for patient-reported outcome measures used in patient-centered outcomes and comparative effectiveness research. Qual Life Res.

[10] Strada L, Vanderplasschen W, Buchholz A, Schulte B, Muller AE, Verthein U, Reimer J (2017). Measuring quality of life in opioid-dependent people: a systematic review of assessment instruments. Qual Life Res.

[11] The WHOQOL Group. WHOQOL-BREF: introduction, administration, scoring, and generic version of the Assessment. Geneva: World Health Organization; 1996. p. 18.

[12] Chang KC, Wang JD, Tang HP, Cheng CM, Lin CY (2014). Psychometric evaluation, using Rasch analysis, of the WHOQOL-BREF in heroin-dependent people undergoing methadone maintenance treatment: further item validation. Health Qual Life Outcomes.

[13] van Nieuwenhuizen C, Schene AH, Koeter MW, Huxley PJ (2001). The Lancashire quality of life profile: modification and psychometric evaluation. Soc Psychiatry Psychiatr Epidemiol.

[14] Ventegodt S, Andersen NJ, Merrick J. QOL10 for clinical quality-assurance and research in treatment-efficacy: Ten key questions for measuring the global quality of life, self-rated physical and mental health, and self-rated social-, sexual- and working ability. J Altern Med Res. 2009;1(2):113-22.

[15] Muller A, Skurtveit S, Clausen T (2016). Validating the generic quality of life tool “QOL10” in a substance use disorder treatment cohort exposes a unique social construct. BMC Med Res Methodol.

[16] Lauritzen G, Ravndal E (2004). Introduction of the EuropASI in Norway: clinical and research experiences from a cost-effectiveness study. J Subst Abus.

[17] Ravndal E, Lauritzen G (2004). En prospektiv studie av stoffmisbrukere i behandling i Norge [a prospective study of substance abusers in treatment in Norway]. NORDISK ALKOHOL- & NARKOTIKATIDSKRIFT [Nordic Journal of Alcohol and Narcotics].

[18] Muller A, Skurtveit S, Clausen T (2016). Many correlates of poor quality of life among substance users entering treatment are not addiction-specific. Health Qual Life Outcomes.

[19] Skjærvø I, Skurtveit S, Clausen T, Bukten A (2016). Substance use pattern, self-control and social network are associated with crime in a substance-using population. Drug Alcohol Rev.

[20] Lei PW, Wu Q (2007). Introduction to structural equation modeling: issues and practical considerations. Educ Meas Issues Pract.

[21] Kline RB (1998). Measurement models and confirmatory factor analysis. Principles and practices of structural equation modeling..

[22] Byrne B (2016). Structural equation modeling with AMOS: basic concepts, applications, and programming.

[23] Feelemyer JP, Des Jarlais DC, Arasteh K, Phillips BW, Hagan H (2014). Changes in quality of life (WHOQOL-BREF) and addiction severity index (ASI) among participants in opioid substitution treatment (OST) in low and middle income countries: an international systematic review. Drug Alcohol Depend.

[24] Karow A, Verthein U, Pukrop R, Reimer J, Haasen C, Krausz M, Schafer I (2011). Quality of life profiles and changes in the course of maintenance treatment among 1,015 patients with severe opioid dependence. Subst Use Misuse.

[25] Roe B, Beynon C, Pickering L, Duffy P (2010). Experiences of drug use and ageing: health, quality of life, relationship and service implications. J Adv Nurs.

[26] Laudet AB, Morgen K, White WL (2006). The role of social supports, spirituality, religiousness, life meaning and affiliation with 12-step fellowships in quality of life satisfaction among individuals in recovery from alcohol and drug problems. Alcohol Treat Q.

[27] De Maeyer J, Vanderplasschen W, Broekaert E (2009). Exploratory study on drug Users' perspectives on quality of life: more than health-related quality of life?. Soc Indic Res.

[28] Tracy EM, Laudet AB, Min MO, Kim H, Brown S, Jun MK, Singer L (2012). Prospective patterns and correlates of quality of life among women in substance abuse treatment. Drug Alcohol Depend.

