# Supplementary material for: Confirming the factor structure of a generic quality of life instrument among pre-treatment substance use disorder patients
Source: Health Qual Life Outcomes. 2019 May 17;17:84. doi: 10.1186/s12955-019-1152-7 (PMC6525421; doi:10.1186/s12955-019-1152-7)
Supplement: Supplementary file 1 — Table S1 QOL10 item responses among 531 patients entering substance use disorder treatment, N(%) (DOCX 14 kb) [file 12955_2019_1152_MOESM1_ESM.docx]

| **Supplementary Table 1: QOL10 item responses among 531 patients entering substance use disorder treatment, N(%)** | | | | | | |
| --- | --- | --- | --- | --- | --- | --- |
|  | “Very poor” | “Poor” | “Neither good nor poor” | “Good” | “Very good” | Mean (SD) |
| 1 Physical health | 106 (20.0) | 155 (29.2) | 153 (28.8) | 99 (18.6) | 18 (3.4) | 1.56 (1.11) |
| 2 Mental health | 121 (22.8) | 210 (39.5) | 120 (22.6) | 68 (12.8) | 12 (2.3) | 1.32 (1.03) |
| 3 Feel about yourself | 106 (20.0) | 198 (37.3) | 136 (25.6) | 83 (15.6) | 8 (1.5) | 1.41 (1.02) |
| 4 Relationship to friends | 58 (10.9) | 160 (30.1) | 125 (23.5) | 155 (29.2) | 33 (6.2) | 1.9 (1.13) |
| 5 Relationship to partner | 36 (14.2) | 58 (22.8) | 55 (21.7) | 64 (25.2) | 41 (16.1) | 2.06 (1.3) |
| 6 Ability to love | 36 (6.8) | 94 (17.7) | 95 (17.9) | 196 (36.9) | 110 (20.7) | 2.47 (1.19) |
| 7 Sexual functioning | 47 (8.9) | 93 (17.5) | 125 (23.5) | 160 (30.1) | 106 (20) | 2.35 (1.23) |
| 8 Social functioning | 58 (10.9) | 153 (28.8) | 150 (28.2) | 135 (25.4) | 35 (6.6) | 1.88 (1.11) |
| 9 Work ability | 160 (30.1) | 161 (30.3) | 104 (19.6) | 76 (14.3) | 30 (5.6) | 1.35 (1.21) |
| 10 Overall quality of life | 188 (35.4) | 215 (40.5) | 96 (18.1) | 30 (5.6) | 2 (0.4) | 1.56 (1.11) |
