# Supplementary material for: Confirming the factor structure of a generic quality of life instrument among pre-treatment substance use disorder patients
Source: Health Qual Life Outcomes. 2019 May 17;17:84. doi: 10.1186/s12955-019-1152-7 (PMC6525421; doi:10.1186/s12955-019-1152-7)
Supplement: Supplementary file 2 — Table S2 QOL10 item-total correlations (DOCX 14 kb) [file 12955_2019_1152_MOESM2_ESM.docx]

| **Supplementary Table 2: QOL10 item-total correlations** | | | | | | |
| --- | --- | --- | --- | --- | --- | --- |
|  |  | Social domain | | | | |
|  |  | 1 | 2 | 3 | 9 | 10 |
| 1 Physical health |  | 1.000 |  |  |  |  |
| 2 Mental health |  | 0.339 | 1.000 |  |  |  |
| 3 Feel about yourself |  | 0.249 | 0.437 | 1.000 |  |  |
| 9 Work ability |  | 0.141 | 0.332 | 0.309 | 1.000 |  |
| 10 Overall quality of life |  | 0.463 | 0.294 | 0.355 | 0.385 | 1.000 |
|  |  |  |  |  |  |  |
|  |  | Global domain | | | | |
|  |  | 4 | 5 | 6 | 7 | 8 |
| 4 Relationship to friends |  | 1.000 |  |  |  |  |
| 5 Relationship to partner |  | 0.344 | 1.000 |  |  |  |
| 6 Ability to love |  | 0.340 | 0.591 | 1.000 |  |  |
| 7 Sexual functioning |  | 0.369 | 0.381 | 0.379 | 1.000 |  |
| 8 Social functioning |  | 0.376 | 0.454 | 0.469 | 0.409 | 1.000 |
